# Adapting the Aerogen Mesh Nebulizer for Dried Aerosol Exposures Using the PreciseInhale Platform

**DOI:** 10.1089/jamp.2019.1554

**Published:** 2020-04-02

**Authors:** Per Gerde, Mattias Nowenwik, Carl-Olof Sjöberg, Ewa Selg

**Affiliations:** ^1^ Inhalation Sciences Sweden AB, Huddinge, Sweden.; ^2^ Institute of Environmental Medicine, Karolinska Intitutet, Stockholm, Sweden.; ^3^ Flexura AB, Upplands Väsby, Sweden.

**Keywords:** Aerogen, aerosol drying, mesh nebulizer, PreciseInhale

## Abstract

***Background:*** Many substances used in inhalation research are water soluble and can be administered as nebulized solutions. Typical examples are therapeutic, small-molecular agents, or macromolecules. Another category is a number of water-soluble agents used for airway diagnostics or disease modeling. Mesh nebulizers have facilitated well-controlled liquid aerosol exposures. Meanwhile, a benchtop inhalation platform, PreciseInhale, was developed for providing small-scale, well-controlled aerosol exposures in preclinical configurations. The purpose of the current research was to adapt the Aerogen mesh nebulizer to work within the PreciseInhale system for both cell culture and rodent exposures.

***Methods:*** The wet aerosols produced with the Aerogen Pro nebulizer were dried out in an aerosol holding chamber by supplying dry carrier air, which was provided by passing the incoming ambient air through a column with silica gel. The nebulizer was installed in an aerosol holding chamber between an upstream flow-rate pneumotach and a downstream aerosol monitor. By pulsing, the nebulizer output was reduced to 1%–10% of continuous operation to better match the exposure ventilation requirements. Additional drying was obtained by mantling the holding chamber with dried paper.

***Results and Conclusions:*** The nebulizer output was reduced to 3–30 μL/min and dried out before reaching the *in vitro* or *in vivo* exposure modules. Using solute concentrations in the range of 0.5%–2% (w/w), dried aerosols were produced with a mass median aerodynamic diameter of 1.5–2.0 μm, compared to the 4–5 μm droplets emitted by the nebulizer. Controlling the Aerogen nebulizer under a reduced output scheme within the PreciseInhale platform gave two major advantages: (i) by reducing aerosol output to better match exposure flow rates of single rodents, increased airway deposition yields were obtained in a range of 1%–10% relative to the nebulized amount of test substance and (ii) shrinking aerosol particle sizes through drying improved the peripheral lung deposition of test aerosols.

## Background

Solid dry powders or sprayed liquid solutions or dispersions are the two major physical forms, in which aerosols for research are administered either to humans in the clinic or to preclinical test models. Each method has its clear merits. Dry powder is the most desirable form for inhalation drugs due to stability and ease of use, and it is also a common form in which aerosol air pollutants appear at exposures. However, in the early discovery phase of inhalation drug development, bypassing the powder formulation step can be of great advantage. If the test substance is water soluble enough to be delivered as true aqueous solutions, the convenience and ease of use of modern mesh nebulizers merits the option of also being able to choose liquid aerosol exposures.

Mesh nebulizers such as the Aerogen (Aerogen, Ltd., Galway, Ireland) have been in use for over a decade,^(1)^ both in the clinic and in various preclinical exposure uses with larger animals,^(2)^ smaller animals/rodents,^(3,4)^ and cell culture models.^(5)^ It has a high aerosol output in the upper respirable size range^(6)^ that is suitable to expose single subjects with larger lungs or multiple rodents at a time. The particle size distribution of generated aerosols from mesh nebulizers has a complex relationship to composition parameters of the feed liquid such as electrolyte concentration^(7)^ and viscosity.^(8)^ With regard to viscosity, the Aerogen is limited to handling approximately a doubling, to 2 cP, compared to deionized water.^(8)^ During optimal conditions, mesh nebulizers typically generate aerosols down into the upper respirable size range, or 4–5 μm mass median aerodynamic diameter (MMAD).

Because of the larger particle sizes generated, attempts have been made to shrink the particle size by drying the liquid aerosol before inhalation to improve particle deposition in the deep lung compared to deposition of the nominal particle sizes emitted by the nebulizer. If processing the entire output from the Aerogen for size reduction, the required equipment becomes fairly large.^(9)^ Moreover, for exposure of single rodents, the aerosol production rate is too high to match typical rodent ventilation rates, giving a low substance utilization.^(10)^

The PreciseInhale^®^ exposure platform provides small scale benchtop exposures of a number of exposure modules from cell cultures to rodents (either intratracheally intubated or nose-only), to larger lungs such as dogs. The purpose is to provide a seamless switch between the exposure models giving them near identical aerosol exposure conditions. Exposures are provided to individual animals allowing controlled dosing of smaller lungs based on the actual aerosol concentration and the ventilation rate of the exposed animal.^(11)^

Because liquid aerosols constitute an even more dynamic exposure atmosphere than dry powders, there is need for a better understanding of the process parameters controlling the aerodynamic properties of nebulized aerosols at the point where they actually reach the exposure zone. With this in mind, the aim of the current research was to adapt the Aerogen Pro mesh nebulizer for integrated use within the PreciseInhale platform. The adaption was accomplished by reducing aerosol output rate to be suitable primarily for single rodent- or cell culture exposures and by shrinking the liquid aerosol droplets by supplying a flow-matched dry air stream to the exposure line.

## Materials and Methods

The Aerogen Pro was integrated in the PreciseInhale system. This allowed utilizing some important service functions of the exposure platform. The nebulizer was built into the exposure line between an upstream flow meter (pneumotach) and a downstream aerosol monitor instrument located immediately before the choice of exposure module.

The nebulizer head discharges into a 200-mL aerosol holding chamber continuing past a light dispersion instrument (Casella Microdust Pro, United Kingdom) onto the chosen downstream exposure module. The aerosol holding chamber has two important functions: First, to merge the aerosol pulses from the mesh nebulizer into a more continuous aerosol stream. Second, the holding chamber allows for reducing the particle size of the generated aerosol through drying. Dry exposure carrier air then enters the aerosol holding chamber through an annular perforated diffusor plate surrounding the Aerogen output pipe. During exposures of a cell culture module or any of the rodent exposure modules, carrier air is drawn with a downstream vacuum pump (Vaccubrand ME-1, Germany) at constant flow rate controlled with a precision needle valve (Brooks). Under such arrangement, the ventilation pattern of exposed rodents will be superimposed on the constant airflow rate drawn by the vacuum pump and will be registered by the upstream pneumotach. The goal for further development is to use the ventilation data, in combination with the signal from the aerosol monitor instrument for active dose control of the aerosol, in the same manner as in the PI (PreciseInhale^®^) dry powder application.^(11)^ Drying of carrier air was accomplished with 800 mL silica gel in a drying column located upstream of the flow meter. After drying, the carrier air was filtered through a 65 mm filter pad (PARI, Germany) placed at the drying column exit to prevent silica gel dust from entering the exposure line.

With the strategy of exposing one small animal at a time to achieve an active dosing of the individuals, a problem arises with the Aerogen having a too high aerosol output for single rodent exposures.^(3)^ Theoretical calculations indicate that an optimal air flow rate for delivering an exposure aerosol to a rodent, without suffering significant rebreathing of exhaled aerosol or wasting too much test material, lies around 2.5 times the ventilation rate of the animal.^(12)^ At full output from the nebulizer consuming 300–400 μL/min of feed liquid, the kinetic energy of the produced aerosol creates a highly mobile plume of entrained air and nebulizer spray, with a stopping distance in surrounding air several 100 mm downstream of the nebulizer exit pipe. This causes a mismatch when trying to deliver the aerosol into a carrier airstream that has been adjusted according to the ratio described above. Instead, the majority of the produced aerosol bypasses the breathing zone causing excessive waste of exposure material. Therefore, the Aerogen output was reduced by pulsing the operation to a fraction of continuous use, adjustable from typically 1% to 10% of the full output. This corresponds to a consumption of feed liquid at about 3–30 μL/min, instead of the full output given above.^(8)^

Applying an output reduction scheme to the Aerogen for better matching the requirement of single rodent- or cell culture exposures provided some challenges, but several advantages in addition to the obvious more efficient use of the test material; the flooding of the exposure module with excess liquid was avoided and the aqueous aerosol output relative to the superimposed carrier air flow rate was such that the particle size distribution of generated aerosol could be rapidly reduced by drying. With the low liquid aerosol output/carrier air flow ratio used here, aerosol particle size will be very sensitive to the humidity of the incoming carrier air.^(13)^ If applying completely dry carrier air at the inlet, the aerosol droplets will dry out and shrink toward the size of the corresponding dry residue aerosol. According to a standard Mollier diagram, completely dry air will be humidified to saturation at a temperature of 22°C when 20 μL water has been evaporated into 1 L of dry air. This represents the maximum evaporative capacity for pure water by dry carrier air at room temperature. However, when having a solute load in the feed solution that often is hygroscopic, it is recommended to supply an excess of dry carrier air to the exposure stream to maintain a positive driving force for the drying process troughout the holding chamber. One challenge, arising from the operation of the nebulizer at a substantially reduced output is, that with ambient pressure in the liquid container, the nebulizer function may cease, because feed liquid is seeping through the downward facing mesh. This problem can be prevented by sealing the liquid feed container with an air tight lid and applying a slight vacuum of typically −10 to −20 mm H_2_O to the container lid using a vacuum eductor.^(14)^

In the current experiments, an exposure/carrier air flow rate of 400 mL/min was chosen to bring the produced aerosol from the nebulizer to exposure, which is suitable for single rodent- or cell culture exposures. At this flow rate, a nebulized stream of 8 μL/min water can be completely evaporated. A first effect of the evaporated water will be to add and increase the average volume of the humidified air with ∼2.5%, when reheated by the conduit walls to the temperature of the incoming air. However, because the nebulizer is used with pulsed operation, a second effect of the rapidly evaporating water, coinciding with each spray pulse, will be a rapid and transient cooling, causing a brief contraction of the air between the pneumotach and the downstream vaccuum source. This cooling contraction will show up as a positive spike in the superimposed flow rate curve. By evaporating a delivered solute aerosol toward dryness, it is possible to reduce the particle size distribution to more suitable values for achieveing a more peripheral deposition of test aerosols in the respiratory tract of exposed rodents.

In the current study, five different feed solutions were used to investigate the properties of the system:

I . A low-salt solution of sodium chloride, 0.05% (w/w), in water to generate an almost solute-free water aerosol.
I I. A 2% solution of terbutaline sulfate (w/w) in a 1:20 diluted solution of phosphate-buffered saline (PBS) to represent an asthma drug of high water solublility.
I II. A 0.5% solution of terbutaline sulfate (w/w) in a 1:20 diluted solution of PBS to represent an asthma drug of high water solublility.
I V. A 1.5% solution of naratriptan (w/w) in a 1:20 diluted solution of PBS, representing a substance used near its solubility limit.
V . A 2% solution of methacholine chloride (w/w) in a 1:20 diluted solution of PBS to represent an important substance for testing airway reactivity.

The low-salt solution was used to investigate the evaporative properties of the nebulizer module when the amount of dry residue in the aerosol is very low. The asthma drug terbutaline sulfate was used as a model substance of higher solubility. It has an aqueous solubility of 213 mg/mL (DrugBank). Naratriptan, a neurologically active substance with an aqueous solubility of 35 mg/mL (DrugBank), was chosen as a test substance having a solubility just above the concentrations chosen in the current Aerogen tests. Methacholine, with an “unlimited solubility” in water,^(15)^ was chosen as a representative of the numerous substances that are used to diagnose airway disease in laboratory animals.

**FIG. 1.**
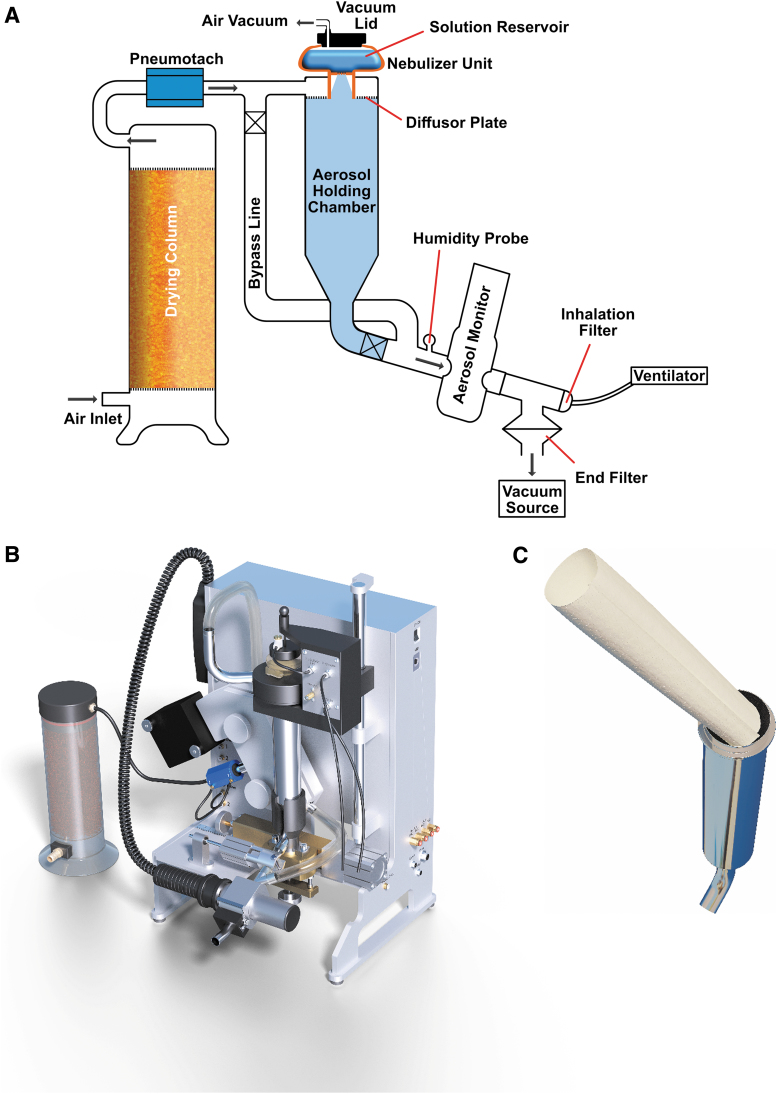
**(A)** The principle of operation of the nebulizer unit. The Aerogen Pro mesh nebulizer was connected to the PreciseInhale platform in an aerosol holding chamber between an upstream flow rate pneumotach and a downstream aerosol monitor and vacuum source. The liquid reservoir of the nebulizer was sealed with a vacuum lid. For the humidity measurements, the humidity probe was installed at a branch tube immediately before the aerosol light dispersion instrument. The incoming air was dried and filtered in a drying column with silica gel. The exposure module was located immediately downstream of the aerosol monitor. **(B)** A picture of the PreciseInhale platform with the liquid aerosol generator properly installed. Vacuum is provided by a vacuum eductor contained in the nebulizer holding assembly and regulated by the needle valve in the control box next to the nebulizer head. **(C)** The aerosol holding chamber with a scrolled drying paper to be inserted. This drying mantle consists of a filter paper (Munktell Filter, Quality 1600, Sweden).

To further increase the aerosol drying capacity of the nebulizer module, the aerosol holding chamber was covered with a humidity absorbing paper mantling inside (Fig. 1C). Through this optional addition of drying capacity, higher output of dried aerosols can be produced for shorter time periods.^(16)^ A similar strategy with activated carbon as an adsorbent in a perforated wall tube reactor has also been used to remove organic vapor solvents from an aerosol exposure flow before reaching the breathing zone.^(17)^ Paper cellulose without surface-coating materials has a sigmoidal moisture isotherm typically absorbing 7% water (w/w) at 50% relative humidity in air.^(18)^ To qualify as a suitable drying mantle, a paper sheet should be thick enough to provide weight and have a porous structure to increase drying speed.^(18)^ In the current study, we chose a filter paper quality for the mantle with a dry weight of 1.6 g (Munktell Filter, Quality 1600, Sweden). During use, the paper must be regularly replaced with a fresh dry paper. A prerequisite for altering the aerosol particle size by coating the cylindrical aerosol holding chamber walls with a hygroscopic material during laminar flow is that the residence time of the aerosol is not shorter than diffusional equilibration time for water vapor in the cross-section of the aerosol holding chamber. At a diameter of 36 mm, the diffusional equilibration time of the cylindrical holding chamber during laminar air flow can be expected to be around 10 seconds.^(19)^

Several series of tests were conducted, to assess the performance of the Aerogen nebulizer unit, using the PreciseInhale platform.

In the first series of tests, the aerosol output of the low-salt solution and 2% terbutaline was measured at different outputs of the nebulizer in percent of continuous generation. The frequency of the operation cycle was set to one pulse per second. The tests were performed by running the nebulizer for 10 minutes at each output setting and then determining the weight loss of the nebulizer head.

In the second series of tests, the carrier air humidity and temperature were measured with a humidity probe (Honeywell HIH-4000 Series) in an aerosol free end pipe branching off upward from the exposure line (Fig. 1A). Aerosols were generated with the low-salt solution and with the 2% terbutaline with either dry air or with dry air plus a drying paper mantle. Aerosols were generated at different low output settings for 10 minutes, and the humidity was registered for up to 15 minutes.

In the third series of tests, the particle size distribution of generated aerosols was established using either dry or humidified inlet air and at different output rates from the nebulizer. In the chosen range of low aerosol output rate, the main method to preserve the particle size distribution of the dynamic aerosols through the cascade impactor is to use dilution air of the same humidity in the cascade impactor as in the carrier air exiting the exposure line of the PreciseInhale system during aerosol generation. Correctly measuring particle size distribution of liquid aerosols is a known problem where different correction procedures have been proposed.^(20)^ Therefore, the particle size distribution was determined with a eight-stage Marple cascade impactor^(21)^ at a carrier air flow rate for the aerosol of 400 mL/min with subsequent dilution to a flow rate of 2 L/min through the impactor using a dilution tunnel at the impactor inlet. To maintain the aerosol particle size close to the generated size, the supplied dilution air was kept near the same relative humidity as the aerosol exiting the exposure line. For size detemination of liquid or humid powder aerosols on the Marple impactor, it is necessary to use metal plates on the stages instead of filter paper. Otherwise, the size distribution will be erroneously fine because of rapid aerosol droplet drying when the aerosol passes along the filter paper stages of the impactor. The dry aerosols were also deposited on microscope coverslip glasses and then immediately kept in a desiccator for later investigation using scanning electron microscopy (SEM). The aerosol was then deposited on the coverslips without dilution air in a deposition chamber similar to the one developed for the Dissolv*It* system.^(22)^

In using the PreciseInhale system for active dosing of any exposure module, the aerosol monitor signal must be calibrated against the gravimetic amount of aerosol causing the detected signal. This is done by arranging phantom filter exposures where total filters are placed in the exposure zone and ventilated at flow rates similar to that of the exposure subjects. For rodent phantom exposures, two filters are used; one larger 25 mm GF/A (Whatman) end filter immediately downstream of the exposure port collecting excess and exhaled aerosol at a constant superimposed flow rate and a smaller 6 mm GF/F (Whatman) inhalation filter placed at the rodent inhalation zone of either intratracheally intubated or nose-only rats. This latter filter was ventilated at typical rodent breathing patterns (Fig. 1A).^(11)^ Following such phantom exposures, a substance correlation factor can be defined correlating the optical and gravimetric mass flux of aerosol inhaled by the exposure subject. This correlation factor is then used by the software to control the individual exposures in real-time. In the current study, tests were done with phantom filters for both intratracheally intubated- and nose-only exposures. The data can then be used to predict the dosing rates that will be obtained during subsequent *in vivo* exposures.

Theoretical deposition patterns of the three differently sized aerosols of wet naratriptan and two dried terbutaline aerosols in the respiratory tract of the rat during intratracheally intubated- and nose-only exposures were calculated using the Multiple-Path Particle Dosimetry (MPPD) model (Applied Research Association, Albuquerque, NM). Simulations were made for assumed realistic breathing patterns in both modules.

## Results

**FIG. 2.**
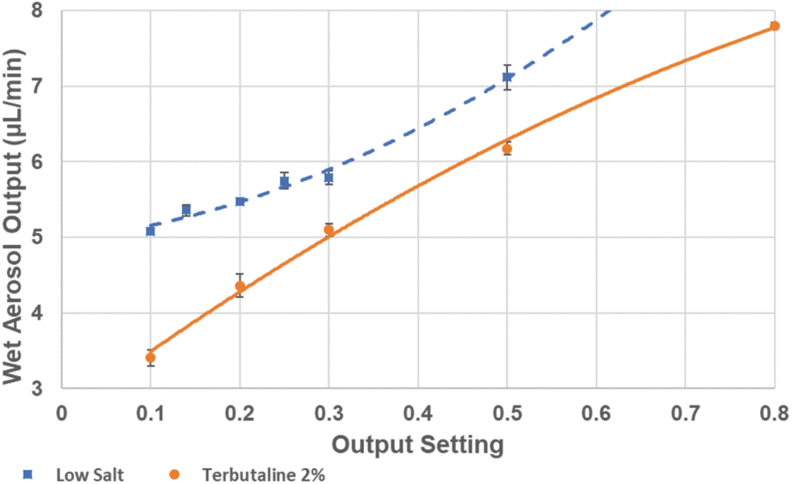
Examples of the wet aerosol production rate from the nebulizer head at different output settings in the lower output range with a low-salt solution and a 2% terbutaline solution. The nebulizer operation was set to one spray pulse of adjustable duration per second. The data are mean ± standard deviation (*n* = 3).

**FIG. 3.**
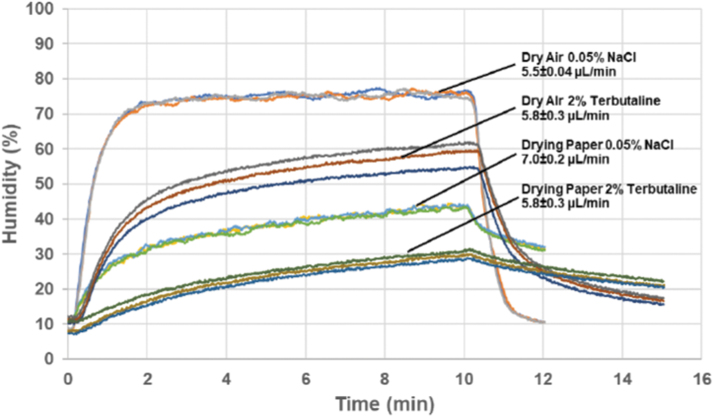
Relative humidity of air exiting the aerosol collection chamber following aerosol generation with different operative settings. These sets of experiments were all performed with dry inlet air (RH <3%) and an aerosolization period of 10 minutes. The displayed sequence was recorded after a 4 minutes startup period. The wet aerosol output from the nebulizer ranged from 5.5 to 7.0 μL/min, and the dry carrier air flow was 400 mL/min. RH, relative humidity.

By pulsing the spray emission, the Aerogen Pro mesh nebulizer was efficiently controlled at a substantially reduced wet aerosol output in a range from 3 to 30 μL/min instead of the 400 μL/min typical of continuous operation. The reduced output better matches requirements for exposing single rodents or respiratory cells cultured at the air–liquid interface. While aerosol output differed between different feed liquid compositions, the nebulizer ran stably down to an output of 3 μL/min for most feed liquids, provided they were not too viscous (Fig. 2). At such low output, the aerosol particle size was easily reduced by supplying dry carrier air to the inlet pneumotach at a flow rate with a capacity to receive humidity in 10–20% excess of what was needed to completely evaporate the generated wet aerosol. For these lower wet aerosol outputs to dry air flow ratios, the drying process was monitored by measuring the air humidity separate from the aerosol particles (Fig. 1A). With the low-salt solution (0.05% NaCl), the air humidity responded rapidly and consistently to the ratio between the fully evaporated wet aerosol output and the flow of dry carrier air. The nebulized spray quickly dried out because of total water evaporation, resulting in an expected relative humidity in the exiting air of 75% at given wet aerosol production rate and dry air flow rate (Fig. 3). Introducing a solute content such as 2% terbutaline with the wet aerosol complicated the dynamics, where air humidity fell below that of the corresponding fully evaporated water content of the feed liquid. Even with the less complicated option of not using drying paper, the air humidity climbed gradually to 60% during the 10 minutes generation period, indicating that the generated particles had a substantial residual humidity at the exit of the holding chamber. Compared to the low-salt solution, air humidity decreased more gradually after nebulization was stopped, which may be caused by fall out of terbutaline from repeated runs coating the holding chamber walls. With drying paper mantling the holding chamber, air humidity was further reduced during aerosol generation, but expectedly, decreased even slower after nebulization was stopped.

**FIG. 4.**
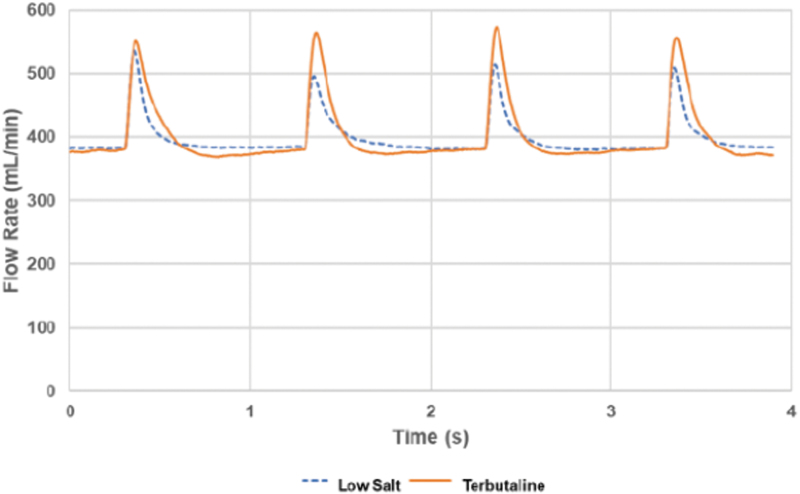
Flow rate monitoring during a 4 seconds period of pulsed aerosol generation with dry inlet air at a flow rate of 400 mL/min. The duration between the flow rate spikes was 1 second, and the most rapid evaporation on each aerosol pulse affecting flow rate lasted less than 0.4 seconds. The low-salt solution was nebulized at 5.5 μL/min, and the 2% terbutaline solution was nebulized at 5.7 μL/min.

The low-salt nebulization into dry carrier air indicated a fast and near complete evaporation in the holding chamber, and this picture was supported by the details of the carrier air flow measurements (Fig. 4). The initial drying of the generated aerosol droplets was very fast as indicated by the inlet air flow spikes detected at each pulse generated by the nebulizer. These air flow spikes, driven by the rapid evaporative cooling of the air in the holding chamber, were noticable for ∼0.4 seconds at each injected aerosol pulse (Fig. 4). If the aerosol nebulization rate was assumed to be constant even during these short duty cycles, each pulse containing about 0.1 μL water should have been emitted within ∼15 ms. It is interesting to note that the air flow spikes were quite similar between the low-salt solution, where a near complete evaporation was detected with the humididy probe, and the air flow spikes generated by the 2% terbutaline solution. The air volume of the integrated flow spikes was actually larger for terbutaline at ∼0.4 mL, compared to ∼0.2 mL for the low-salt solution. This would suggest that the dried terbutaline aerosol consisted primarily of solid residue. However, the full transiency of the flow spikes may be masked by a limited response time of the flow measurement set up, thereby underestimating the full volumetric impact of the carrier air evaporative cooling.

**FIG. 5.**
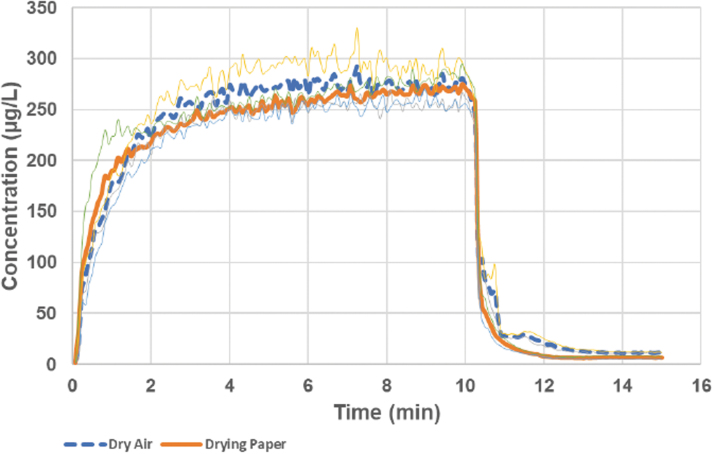
The concentration of dry terbutaline aerosol measured with the light dispersion instrument during the two types of drying strategies: with dry inlet air or with dry inlet air combined with a drying paper mantle in the holding chamber. After each test run, the instrument signal was corrected for the amount of terbutaline collected on the downstream exposure filters and the carrier air flow rate to calculate apparent aerosol concentration. The thin lines indicate ± one standard deviation from three experiments. The wet aerosol output from the nebulizer during the experiments was 5.7 ± 0.3 μL/min, and the carrier air flow rate was 400 mL/min.

Despite the variability in the humidity measurements, the results suggest that, when the drying options were imposed, the generated aerosol droplets will shrink toward the size corresponding to their dry particle residues. As opposed to the wet aerosol, the concentration of dried aerosol from the 2% terbutaline solution could be measured with the aerosol monitor instrument. There was slight difference in results whether just dry air was used or dry air plus drying paper (Fig. 5). When using dry air, the recorded aerosol concentration seemed slightly more stable, whereas when drying paper was used, the measured light scatter from the aerosol continued to increase during the 10 minutes generation period. This is an expected result from the drying paper slowly being saturated with humidity compared to the dry air case, where the holding chamber steel walls were gradually being coated only with deposited solid terbutaline aerosol. The concentrations normalized to the filter collected aerosol immediately downstream of the light dispersion instument were very stable. The calculated substance correlation factors based on aerosol delivered to exposure had a standard deviation between repeated runs of less than 2%, suggesting that the data produced may well be used for active dosing purposes of preclinical exposure models. The amount of dry aerosol collected on a downstream filter in the exposure zone of the PreciseInhale constituted about 85% of the terbutaline emitted by the nebulizer.

The aerodynamic particle size distribution of the generated aerosols, when sized with the Marple cascade impactor, fell into two ranges depending on the two sets of operating conditions (Table 1). Using the aerosol drying options, all three test solutions in a solute concentration range of 0.5%–2% w/w produced aerosols in the range of 1.2 to 2.0 μm MMAD, with narrow size distributions indicated by geometric standard deviations in the range of 1.8 to 2.3. With the drying paper option, the aerosol particle size was maintained around a MMAD of 2 μm for 5 minutes even when the nebulizer output was increased from 6 to 23 μL/min (Table 1). The dose rate could be increased about threefold during such an output increase. In contrast, trying to maintain droplet sizes at the 4–5 μm, they were emitted from the nebulizer head by using humidified carrier air, proved to be more difficult. Only the less soluble naratriptan generated a wet aerosol of 4 μm MMAD at the exit from the holding chamber, whereas the more soluble terbutaline rained out already in the holding chamber, most likely because of droplet size growth.^(13)^ This phenomenon discourages from use of humidified carrier air to maintain liquid aerosol sizes of solutes at their generated particle size when operating the nebulizer at these low wet aerosol output/carrier air flow ratios.

**Table 1.** Aerodynamic Particle Size Distribution of Generated Aerosols as Determined with an Eight-Stage Marple Impactor at a Carrier Air Flow Rate of 400 mL/min

Feed solution	Feed rate (μL/min)	Carrier air status	MMAD (μm)	GSD
Terbutaline 2%	5–7	Humid air	Rain out	—
Terbutaline 2%	5–7	Dry air	1.9 ± 0.1	2.0
Terbutaline 2%	5–7	Drying paper	1.9 ± 0.0	2.0
Terbutaline 2%	21–25	Drying paper	2.0 ± 0.1	1.9
Terbutaline 0.5%	5–7	Drying paper	1.5 ± 0.05	2.1
Naratriptan 1.5%	5–7	Humid air	4.0 ± 0.6	2.3
Naratriptan 1.5%	5–7	Dry air	1.2 ± 0.1	2.1
Methacholine 2%	5–7	Drying paper	1.7 ± 0.1	1.8

Average ± standard deviation (*n* = 3).

GSD, geometric standard deviation; MMAD, mass median aerodynamic diameter.

**FIG. 6.**
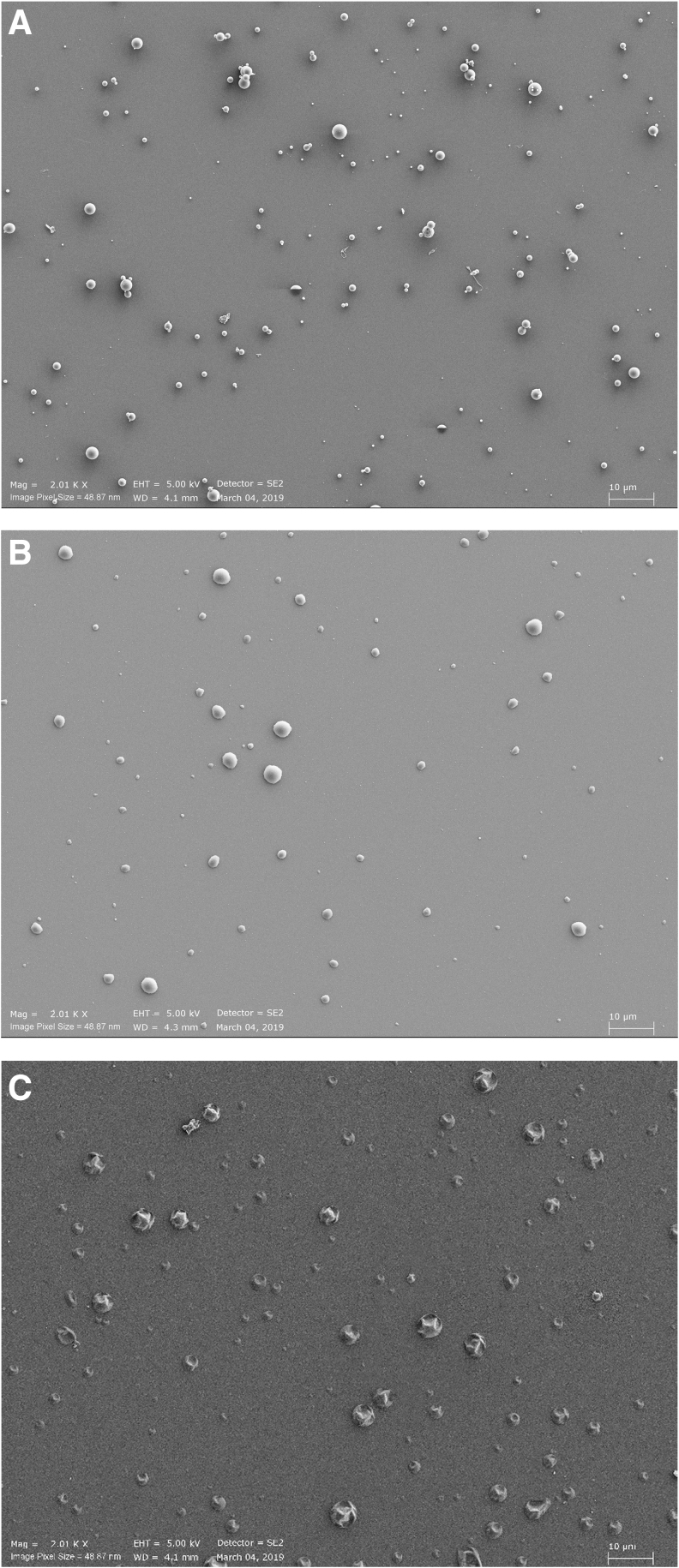
Scanning electron microscopy pictures of generated dried aerosols. **(A)** Terbutaline 2%; **(B)** terbutaline 0.5%; **(C)** methacholine 2%.

Collecting the dried aerosols on coverslip glasses for SEM at the position of the exposure modules indicated that solid particles begin to form, and the deposited particles seem to have sizes corresponding to their aerodynamic sizes, as determined in the cascade impactor (Fig. 6). However, it is likely that additional drying had occurred during dry storage of the collected aerosols before SEM was performed. Because of this variable additional drying, the different solutes created particles with morphologies that varied substantially. The 2% terbutaline solution formed perfect spheres, while 2% methacholine generated particles looking like crumpled spheres (Fig. 6C).

Because the reduced aerosol production rate better matches the laminar flow pattern through the holding chamber and the optimal flow requirements for single rodent exposures, the exposure efficiency will be quite high. With low-static aerosols such as the ones generated in the current study, the fraction of emitted solute that reached the breathing zone lies in the range of 75%–85%. The phantom filter tests simulating rodent inhalation exposures indicated that the amount of substance that was inhaled at the rat nose or at the intubated rat trachea during exposures varied from 22% to 23% of the emitted amount for the nose-only module to 12% of emitted aerosol for the intratrachally intubated module, depending primarily on different ratios of carrier air flow to ventilation rates (Fig. 1A and Table 2). Adding simulated airway deposition data for the rat from the MPPD model provided indicative deposition rates of the generated aerosols in the major regions of the rat respiratory tract (Table 3). With the tracheally intubated module, the advantage of bypassing the head airways of the rat is evident from the predicted high deposition efficiencies of all three aerosol sizes. Even with the lower inhaled fraction of aerosol, the overall deposition rate in bronchi plus alveoli in the intracheally intubated module will be ranging from 3% to 6% of emitted aerosol for aerosols ranging from 1.5 to 4 μm MMAD. For the nose-only module, losses in the head airways will be substantial for all generated aerosols, reducing deposition in the lungs to only 1%–4% of the inhaled aerosol mass (Table 3). This constituted only 0.2%–0.9% of the emitted amount from the nebulizer. However, it should be noted that shrinking of the aerosol size through drying during nose-only exposures increases lung deposition efficiencies threefold to fourfold compared to the wet aerosol alternative.

**Table 2.** Filter Exposures Predicting Aerosol Inhalation and Deposition Rates of 2% Terbutaline During Simulated Intratracheal and Nose-Only Rat Exposures

Exposure mode	Wet aerosol output (μL/min)	Inhalation rate (μg/min)	Head deposition (%)	Head deposition rate (μg/min)	Lung deposition (%)	Lung deposition rate (μg/min)
Intratracheal	5.5	14	N/A	N/A	30	4
Intratracheal	25	53	N/A	N/A	31	16
Nose-only	5.5	25	64	16	22	0.5
Nose-only	25	98	67	65	21	2

The superimposed carrier air flow rate was 400 mL/min. Intratracheal inhalation exposures was simulated at 50 breaths per min and a tidal volume of 1.5 mL, and, the nose-only exposures was simulated at 160 breaths per minute with a tidal volume of 1.1 mL. Percent deposition of inhaled aerosol was calculated using the MPPD model (v.2.11).

MPPD, Multiple-Path Particle Dosimetry.

**Table 3.** Regional Airway Deposition of Some Generated Wet and Dried Aerosols Following Intratracheal- and Nose-Only Exposures in the Rat

Exposure aerosol	Intratracheal exposures			Nose-only exposures			
	Total (%)	Bronchi (%)	Alveoli (%)	Total (%)	Head (%)	Bronchi (%)	Alveoli (%)
4.0 μm naratriptan	48	25	23	85	84	0.5	0.5
1.9 μm terbutaline	30	9	21	67	64	1.5	1.5
1.5 μm terbutaline	25	7	18	56	53	1.5	1.5

Simulations were made with the MPPD model (v.2.11) for intratracheal exposures with a breathing rate of 50 breaths per minute at 1.5 mL tidal volume, and for nose-only exposures with a breathing rate of 160 breaths per minute at a tidal volume of 1.1 mL. All deposited fractions are expressed as percent of inhaled.

## Discussion

The Aerogen Pro mesh nebulizer combined with the dynamic vacuum system for the liquid container was integrated into the PreciseInhale platform to allow a controlled generation and delivery of nebulized aerosols. This gave several advantages: (i) by applying a slight vacuum to the nebulizer liquid container, the output from the nebulizer could be substantially reduced to better match the ventilation rates of rodents, without experiencing liquid seeping through the mesh thereby interrupting aerosolization. (ii) When exposing rodents one at a time, the substance utilization was significantly improved. (iii) The substantially reduced nebulizer output permitted drying out the aerosol to achieve smaller particle size distibutions, promoting higher peripheral lung deposition. This is favorable, particularly for rodent nose-only inhalation exposures. (iv) Dried aerosols allowed using the in-line aerosol monitor for concentration measurements. This constitutes a basis for further development, the goal of which is combining the aerosol concentration measurement with continuous ventilation measurement of the exposed animal for active dosing purposes that will give a significant improvement in the precision of dosing.

In evaluating performace of the low output nebulizer system, it is important to understand the relationship between the rate of wet aerosol produced by the mesh nebulizer and the carrier air flow transporting it to exposure. If the nebulizer head is placed in the exposure holding chamber with both inlet- and outlet ports left open to ambient air, the nebulizer, if operated at full output, drives an aerosol flow through the holding chamber with a volumetric flow rate of ∼1400 mL/min, because of entrainment of air by the nebulizer droplet kinetic energy. At the nominal feed liquid consumption rate of 400 μL/min, the theoretical liquid aerosol concentration would be on the order of 200–300 mg/L if ideal mixing of air and aerosol occurred. However, the high level of aerosol losses that are typically observed at this high output rate is likely caused both by the induced flow rate being too high to match the lower ventilation rates of single rodents, and by the high mass density of the exposure aerosol giving the observed large losses of exposure material both bypassing the nose and depositing on container walls. The latter phenomenon has been described as condensation or rain out when running the nebulizer at high output into a confined exposure container.^(23)^ The rain out process is also likely enhanced by the coalescence of primary droplets into more rapidly impacting/sedimenting larger droplets in the very concentrated and turbulent aerosol plume generated. It is worth to note, however, that at the full wet aerosol output, the humidity in the carrier air of such aerosols should attain equilibrium with the droplets undergoing only minor changes in droplet sizes, irrespective of the carrier being supplied dry or humidified. So hygroscopic growth should not be a factor in the large aerosol losses from rain out as observed when the nebulizer is operated under such high output conditions.

This changes dramatically when an output reduction scheme for the nebulizer is imposed on the system. The rapid droplet drying observed as a transient evaporative cooling of the incoming dry carrier air, which lasts for ∼0.4 seconds at each injected aerosol pulse (Fig. 3B), indicates that the radial mass transfer of water vapor between the droplets and surrounding carrier air is much faster than the typical 30 seconds residence time of the aerosol in the holding chamber before exposure. While this rapid attainment of droplet to air equilibrium has currently only been detected when transport occurs from droplets to dry air, there is reason to assume that a fast radial transport can also occur in the opposite direction when the air has been humidified to levels higher than the droplet/air equilibrium, even if the gradient is substantially smaller. Such a phenomenon could explain the droplet growth and rain out in the holding chamber of the more soluble compound terbutaline, while the lower solubility naratriptan maintained droplet sizes close to those generated by the nebulizer for it to be sized in the cascade impactor (Table 2). It is likely that the equilibrium humidity around droplets of the more soluble terbutaline should be lower than for less soluble naratriptan, creating the difference in behavior. Nevertheless, for us, the result is a caution against at all using humidified carrier air for maintaining aerosol droplet sizes close to the sizes generated, when the nebulizer module is operated at the current low wet aerosol output/carrier air flow ratios and due to the fact that the particle size distribution of the typical wet aerosol generated is already straddling the upper limit for being optimally effective respirable aerosols.

In the dynamic nebulizer aerosols, several mechanisms can be expected to either increase or decrease the aerodynamic particle size. The liquid aerosol droplets may shrink in size because of water evaporation into a carrier air drier than the equilibrium level. Liquid aerosol droplets can also grow in size either because of condensation from carrier air more humid than the equilibrium value at the droplet surface, or from droplets colliding and coalescing into larger sizes. In the dry aerosols, particles may agglomerate to larger sizes particularly if the aerosol has a high electrostatic charge. Static aerosols may also face substantial losses to interior equipment surfaces. This has not been the case with the currently tested substance selection, neither with nor without drying paper. For static aerosols, wall losses with paper may possibly be higher early on before the cellulose becomes hydrated. However, this warrants further study.

Mitigating these drawbacks align well with the intent of drying out the aerosol before exposures and calls for operating the nebulizer in the range of 1%–10% capacity instead of its full output when used within the PreciseInhale platform for single rodent- or cell culture exposures. Figure 2 shows the relationship between the setting of duty cycle length and the feed liquid consumption rate. At setting 0.5, the nebulizer emits 5–6 μL/min liquid aerosol. At a suitable rodent exposure flow rate of 400 mL/min, the wet aerosol concentration will be around 40 mg/L, which after drying will drop to 0.25 mg/L aerosol dry weight (Fig. 4). This still provides a good dosing rate of potentially excipient-free substance for rodent inhalation exposures, and is not far behind the DustGun dry powder generator of the PreciseInhale platform, yet, substantially better than typical dose rates of excipient mixed formulations used with Wright dust feeders in nose-only tower exposures.^(11)^ Furthermore, adding the drying paper to the holding chamber permits a further threefold increase in dried aerosol output for periods of up to 5 minutes without changing paper (Table 1), which can be an advantage when a higher dose rate is desired for a brief period.

It is most likely the secondary air entrainment flow patterns established around the nebulizer head when operated at full output that causes the large substance losses when connected for single rodent exposures in confined spaces. This phenomenon is sometimes labeled condensation,^(23)^ but is more likely caused by aerosol coalescence, impaction, and sedimentation of larger aerosol droplets generated in the local flow patterns of highly concentrated aerosol established around the nebulizer head. At a substantially reduced output the generated aerosol can instead be easily contained within a carrier airstream optimally matched for either exposing single rodents without rebreathing problems or for not bypassing the animal with too much aerosol.^(12)^ Using this option in the current set up, the lung deposition efficiency for typical respirable size aerosols in the intratracheal inhalation module can be expected to range up to 10% of the amount of test substance consumed by the nebulizer. This is between one and two orders of magnitude better than when using the Aeroneb in a single rodent setting at full aerosol output.^(23)^ One particularly attractive opportunity this provides is to test water-soluble compounds in early development, including macromolcules, using inhalation exposures with respirable aerosols and under good dosing control when test materials are available only in the tens of milligram range.

Having the option of drying out aerosols generated by the Aerogen mesh nebulizer is a particular advantage during use in-line with either nose-only or intratracheal inhalation exposures in rodents. In rats by shrinking the aerosol MMAD from 4 to 1.5 μm, the over-all respiratory tract deposition will not change so much, but aerosol penetration and deposition in the peripheral lung will improve substantially (Table 3). The nasal airway deposition during nose-only exposures will be reduced from 84% to 53%, while the fraction of the deposited aerosol that will reach the brochoalveolar region will increase from 1% to 4% of the inhaled aerosol.^(24)^ It must also be kept in mind that the deep lung deposition of some dried aerosols may be further increased by hygroscopic growth of the aerosols in the deeper lung.^(25)^

The nebulizer aerosol alternative is particularly convenient for substrates with an aqueous solubility exceeding ∼2% (w/w), where no extra excipients or surfactants are necessary to create an aerosolizable feed solution.^(26)^ Many of such test substances are hygroscopic and can be difficult to store and generate as dry powder aerosols. The water-soluble therapeutic substances are of obvious interest to administer with this method, both small-molecular ones and the growing group of macromolecules that are now being considered as candidate drugs.^(27)^ Another large group of substances where in-line drying of nebulizer aerosols can be useful is the variety of reasonably water-soluble compounds that are used to induce and titrate airway hyperresposiveness in preclinical rodent models, such as lipopolysaccarides,^(28)^ house dust mite extracts, adenosine,^(29)^ ovalbumine, and the methacholine, tested here.^(30–32)^ Another pretreatment procedure where inhalation of highly respirable aerosols may improve outcome accuracy is with rodent fibrotic models using bleomycin.^(33,34)^ Providing these substances to the lungs as respirable aerosols with higher precision, instead of being instilled as liquids or insufflated as powders to the test subject lungs, may improve the statistical resolution of subsequent experiments. There are preliminary data suggesting that improving respirable aerosol dosing with airway challenge substances such as methacholine will improve the precision in subsequent pharmacodynamic read outs.^(35)^

In conclusion, incorporating the Aerogen mesh nebulizer into the PreciseInhale exposure platform complements the two previous aerosol sources of micron-size dry powders and clinical inhalers with the third option of generating respirable dried aerosols from most water-soluble compounds in a small-scale preclinical setting.

## Notes

Reviewed by:

Lea Ann Dailey

John Dennis
